# Predictors of Psychiatric Emergency Department Visits Following Inpatient Discharge: Secondary Analysis of a Stepped-Wedge Cluster Randomized Trial

**DOI:** 10.2196/79184

**Published:** 2026-03-30

**Authors:** Wanying Mao, Reham Shalaby, Ernest Owusu, Hossam Eldin Elgendy, Belinda Agyapong, Pierre Chue, Peter H Silverstone, Andrew J Greenshaw, Xin-Min Li, Ejemai Eboreime, Wesley Vuong, Arto Ohinmaa, Frank P MacMaster, Vincent Israel Opoku Agyapong

**Affiliations:** 1 Department of Psychiatry, Faculty of Medicine & Dentistry, University of Alberta, Edmonton, AB, Canada; 2 Department of Psychiatry, Faculty of Medicine, Dalhousie University, Abbie J Lane Building 5909 Veterans Memorial Lane, Halifax, NS, B3H 2E2, Canada, 1 780-215-7771, 1 902-473-4887; 3 Addiction and Mental Health Services, Alberta Health Services, Edmonton, AB, Canada; 4 School of Public Health, University of Alberta, Edmonton, AB, Canada; 5 Department of Psychiatry, Cumming School of Medicine, University of Calgary, Calgary, AB, Canada

**Keywords:** psychiatric discharge, emergency department visits, transitional care, supportive text messaging, peer support, mental health

## Abstract

**Background:**

The period following discharge from psychiatric inpatient care represents a critical transition phase marked by heightened vulnerability to relapse, including increased risks of emergency department (ED) utilization. Understanding the risk factors for ED utilization after hospital discharge will help identify individuals who should be targeted for enhanced follow up care in the community.

**Objective:**

This study aimed to examine the sociodemographic and clinical factors associated with psychiatric ED utilization within six months of discharge from inpatient psychiatric care among individuals assigned to different postdischarge interventions. The goal is to identify high-risk groups to inform targeted follow up strategies and enhance transitional care planning.

**Methods:**

This study analyzed secondary data from a pragmatic stepped-wedge cluster-randomized trial which recruited patients across ten health care sites in Alberta, Canada, from March 2022 to February 2024. For the primary study, a total of 1098 psychiatric inpatients were allocated to one of three post-discharge conditions: treatment as usual (TAU), SMS, or SMS plus peer support (SMS+ PS). Sociodemographic and clinical data were collected at discharge. ED visits 6-months postdischarge were recorded. *χ*^2^ tests identified variables associated with ED utilization. Significant predictors were entered into a logistic regression model to determine adjusted odds ratios (ORs) and 95% CIs.

**Results:**

Of the 1098 participants, demographic and clinical variables were examined for association with mental health ED visits at 6-months post discharge. Univariate analysis identified six significant predictors: age, ethnicity, relationship status, employment, housing status, and prior ED use. Logistic regression analysis identified several predictors of mental health ED visits 6-months postdischarge. Compared to participants under 25 years, those aged 26‐40 was less likely to revisit the ED (OR 0.66, 95% CI 0.46‐0.95), as were those over 40 years (OR 0.58, 95% CI 0.37‐0.92). Individuals identifying as mixed or other ethnicity were less likely than White people to return to the ED (OR 0.52, 95% CI 0.28‐0.96). Unemployed participants had higher odds of ED use than those employed (OR 1.66, 95% CI 1.18‐2.34). Prior ED attendance was the strongest predictor (OR 2.45, 95% CI 1.03‐5.80). Housing status showed varied but nonsignificant effects.

**Conclusions:**

This study highlights key demographic and clinical factors influencing psychiatric ED use following inpatient discharge. The findings emphasize the importance of targeted transitional care interventions, particularly for high-risk groups such as younger, unemployed, and previously ED-utilizing individuals, and support the integration of scalable approaches like SMS and peer support into discharge planning.

## Introduction

The transition period following discharge from psychiatric inpatient care is a critical and vulnerable phase in a patient’s recovery journey. This timeframe is frequently associated with heightened risks of symptom relapse, increased psychiatric emergency department (ED) visits, and hospital readmissions [1,2]. Recently discharged psychiatric patients often encounter considerable difficulties, including inadequate access to appropriate outpatient mental health services, limited social support, and fluctuating symptom severity, which contribute to their increased utilization of emergency mental health care services [3-5]. In Canada, approximately 15%‐25% of psychiatric inpatients are readmitted within 30 days of discharge, and ED revisit rates approach 40% within the first year [6,7]. These repeat encounters expose patients to additional stress, disrupt recovery trajectories, and are consistently linked to poorer long-term outcomes.

Emergency department visits for mental health-related concerns have progressively increased over the past decade, creating substantial burdens on health care systems worldwide [6,7]. Frequent ED presentations not only escalate health care costs but also negatively impact the quality of life and psychological stability of patients and their families [8,9]. System-level impacts include overcrowding in EDs, resource diversion from acute medical emergencies, and increased strain on community-based mental health providers who must accommodate unplanned demand. This illustrates how rising ED visits contribute to bottlenecks across the continuum of psychiatric care. Specifically, in Alberta, provincial reports have noted rising psychiatric ED presentations and high rates of relapse following discharge, underscoring the urgency of identifying context-specific predictors that can inform local service planning [10].

Recent research has focused on identifying factors associated with increased ED utilization post discharge. Sociodemographic factors such as age, ethnicity, employment status, and housing conditions have been found to significantly influence patients’ likelihood of returning to EDs. Clinical predictors include primary psychiatric diagnoses (eg, schizophrenia, personality disorders, substance use disorders), comorbid substance misuse, and high symptom burden at discharge. Social risk factors, including unemployment, unstable housing, and weak social support, also increase vulnerability [7,11,12]. Studies have consistently demonstrated that younger age, unstable housing, unemployment, and severe psychiatric conditions, particularly personality disorders and substance use disorders, are linked to higher rates of repeated ED visits [13-15]. However, despite a growing number of international studies, relatively few investigations have examined these predictors in Canadian settings, and even fewer within the province of Alberta. Given Alberta’s health care delivery structures and recent adoption of digital and peer-based transitional supports, understanding predictors in this setting addresses a critical gap in the literature.

Alongside digital solutions, several nondigital transitional interventions have been trialed internationally to reduce psychiatric readmissions and ED visits. These include assertive community treatment, intensive case management, crisis residential services, and structured follow up appointments scheduled shortly after discharge [7]. While effective in some contexts, such approaches can be resource-intensive and difficult to scale, creating interest in low-cost, technology-enabled alternatives such as supportive text messaging.

To address these challenges, various transitional interventions have been developed, incorporating both technological and peer-support elements designed to enhance outpatient continuity and support [7,16]. Among these, supportive text messaging (SMS) interventions have gained traction due to their accessibility, affordability, and potential to maintain continuous engagement with patients after discharge. In this program, participants receive supportive messages designed by clinicians, containing motivational content, coping strategies, and reminders to adhere to treatment plans. Messages are automated but overseen by mental health professionals to ensure quality and appropriateness. Preliminary evidence suggests that such interventions can effectively reduce feelings of isolation, improve adherence to outpatient care plans, and potentially decrease ED presentations and hospital readmissions [17,18]. Additionally, integrating peer support alongside SMS interventions has been proposed as a promising approach, leveraging lived experiences and empathetic interactions to bolster patient engagement and enhance psychosocial recovery outcomes. In our study, peer supporters were trained individuals with lived experience of mental illness and recovery, offering empathetic, recovery-oriented support via in-person and virtual contact. Despite the promise of these innovative transitional strategies, evidence remains unclear regarding their long-term efficacy in reducing psychiatric emergency visits and promoting sustained clinical improvement. Additionally, questions persist regarding the relative efficacy of standalone SMS interventions compared to combined SMS and peer-support programs in reducing frequent ED presentations. Importantly, prior work has rarely connected the study of predictors of ED use to the context in which these transitional interventions are being implemented, leaving uncertainty about whether high-risk groups identified through predictive modeling are the same groups most likely to benefit from SMS and peer support.

The purpose of this study was to examine the sociodemographic and clinical factors that predict psychiatric ED utilization within six months following discharge from inpatient psychiatric care among participants who were assigned to different treatment groups (treatment as usual, TAU, SMS and SMS+ peer support, PS). By situating this analysis within the broader context of transitional interventions currently being tested in Alberta, the study both addresses a clear research gap and provides evidence to support refinement of local service delivery. By identifying high-risk groups for post-discharge ED visits, the study aims to support the development of targeted follow-up strategies and inform transitional care planning. Findings from this study will help health care providers and policy makers refine and optimize transitional care strategies, ultimately aiming to reduce the burden on emergency psychiatric services and improve patient-centered recovery trajectories.

## Methods

### Study Setting and Design

This study analyzed secondary data from pragmatic stepped-wedge cluster-randomized controlled trial which recruited patients across ten major inpatient psychiatric units in Alberta, Canada, from March 2022 to February 2024. While the primary study is focused on cost effectiveness analysis related to the impact of supportive text messaging with and without peer support interventions on acute health services utilization for patients discharged from inpatient psychiatric hospitals, this analysis focuses solely on identifying the sociodemographic and clinical predictors of psychiatric ED visits within six months following discharge. Participants were randomized into three groups: TAU (control group, standard postdischarge care), SMS (TAU plus daily supportive text messages), and SMS+ PS (TAU plus SMS plus structured peer support). As a secondary analysis, the range of available predictors was constrained by the data collected in the parent trial. Several potentially important clinical and social variables, such as diagnose severity, comorbid substance use, treatment adherence, and outpatient service engagement were not available, limiting the ability to adjust for confounding. In addition, the stepped-wedge design, while pragmatic, introduces potential clustering and time effects (eg, site-level changes, policy shifts) that may influence outcomes independent of individual predictors. These factors should be considered when interpreting causal inferences from this analysis.

### Data Collection and Inclusion Criteria

Recruitment was conducted face-to-face, and nursing managers and staff supported recruitment efforts by identifying eligible patients preparing for discharge from inpatient psychiatric units. Potential participants were provided with detailed study information, including informational leaflets. Interested patients gave written informed consent and completed a self-administered questionnaire on tablet devices. The survey, hosted on REDCap, a secure, web-based platform designed for research surveys and databases [19], collected sociodemographic data (eg, age, gender, ethnicity, relationship status, employment, education, housing status) and clinical characteristics (eg, psychiatric diagnoses at discharge, anxiety, depression, and well-being levels). All participants who consented were included in the subsequent analyses. All sociodemographic and clinical variables used in this analysis were self-reported by participants at the time of discharge; diagnostic categories were confirmed against medical discharge notes when available, and information about ED visits were collected from the records of Alberta Health Service.

#### Inclusion and Exclusion Criteria

Participants were required to have a diagnosed mental health condition, be preparing for discharge from an inpatient psychiatry unit, be aged 18 years or older, and possess a mobile device with an active phone number. They were also required to have the ability to receive text messages, read English texts, and provide written informed consent. Patients planning to travel out of town during the 6-months follow-up period were excluded due to the nature of one of the interventions, a peer support service, which necessitated in-person meetings to provide lived experiences shared by peer support workers. The research team collected participants’ phone and health care numbers as primary identifiers. Operational definitions were applied to key variables to ensure clarity and reproducibility. Housing status was categorized as owning, renting, living with family or friends, or unstable housing (eg, shelter, street, couchsurfing, other). Employment status was grouped into employed, unemployed, student, retired, or other. Prior ED use was defined as any psychiatric ED visit in the six months prior to discharge. These categorizations align with prior studies on ED utilization [20-22].

### Missing Data

We followed an intention-to-treat (ITT) approach. For participants with at least one follow-up assessment, the Last Observation Carried Forward method was used to retain them in the analysis. When later follow ups were missing, the last available data point was carried forward, thereby avoiding excessive attrition and preserving statistical power. No more complex imputation methods (eg, multiple imputation) were applied in order to maintain transparency and interpretability of results.

### Ethical Considerations

This study received ethical clearance from the Health Research Ethics Board of the University of Alberta (Ref # Pro00111459) and operational approval from the regional health authority. All participants provided written informed consent prior to participation. Participants’ confidentiality was protected throughout the study; all identifying information was stored separately from research data and accessible only to authorized research personnel. Participants did not receive monetary compensation for participation.

### Sample Size Calculation

The sample size calculation was based on the 28,571 psychiatric discharges recorded in Alberta in 2018. Using a CI of 95% and a margin of error of ±3%, a minimum required sample size of 1036 participants was estimated. This number was rounded slightly upwards to provide a conservative estimate, ensuring adequate statistical power to detect meaningful group differences and maintain representativeness for the general psychiatric inpatient population [10].

### Outcome Measures

#### Generalized Anxiety Disorder Scale (GAD-7)

Anxiety symptoms were assessed using the Generalized Anxiety Disorder 7-item scale (GAD-7), a validated self-report measure. This scale comprises seven items evaluating the frequency and severity of anxiety symptoms over the preceding two weeks, rated on a 4-point Likert scale (0 = “Not at all” to 3 = “Nearly every day”). Scores range from 0 to 21, with higher values indicating more severe anxiety [23]. The GAD-7 demonstrates strong psychometric validity, including a sensitivity of 89% and specificity of 82% for generalized anxiety disorder at the recommended cut-off score of 10, high test-retest reliability (intraclass correlation, ICC=0.83), and excellent internal consistency (Cronbach α=0.92) [23,24].

#### Patient Health Questionnaire (PHQ-9)

Depressive symptoms were measured using the Patient Health Questionnaire-9 (PHQ-9), a self-administered scale based on Diagnostic and Statistical Manual of Mental Disorders, 4th Edition, DSM-IV criteria for major depressive disorder. It consists of nine items scored from 0 (“Never”) to 3 (“Nearly every day”), yielding total scores between 0 and 27 [25,26]. A score of ≥10 indicates clinically significant depression. Severity categories include mild (5), moderate (10), moderately severe (15), and severe (20). The PHQ-9 exhibits good internal consistency (Cronbach α=0.85), robust convergent validity with related measures, and high sensitivity and specificity (approximately 88%) at the ≥10 cut-off [25-27].

#### World Health Organization-Five Well-Being Index (WHO-5)

The World Health Organization-Five Well-Being Index (WHO-5) assessed participants’ overall mental well-being. Developed by the World Health Organization, this brief measure includes five positively phrased items rated from 0 (“Not at all”) to 5 (“All the time”), standardized to a 0‐100 scale. Scores below 50 indicate poor emotional well-being and necessitate further assessment. WHO-5 shows excellent internal consistency (Cronbach α=.90), strong convergent validity with the PHQ-9 (*r*=–0.73), and high sensitivity (93%) and specificity (83%) for detecting depression [28,29].

#### Brief Resilience Scale (BRS)

Resilience, defined as the capacity to recover from stress and adversity, was assessed using the Brief Resilience Scale (BRS), a validated six-item self-report instrument. Each item measures the ability to bounce back from difficult experiences, rated on a 5-point Likert scale (1 = “Strongly disagree” to 5 = “Strongly agree”). Scores are calculated as the mean of all items, with higher scores indicating greater resilience [30]. The BRS has demonstrated solid psychometric properties, including good internal consistency (Cronbach α=0.80‐0.91) and test-retest reliability (*r*=0.69 over one month). It has shown good construct validity with positive correlations with coping resources and negative correlations with perceived stress and depression, supporting its use in clinical and research contexts [30].

### Statistical Analysis

Statistical analyses were performed using SPSS (version 25; IBM Corp) [31]. Initially, univariate analyses were conducted using *χ*^2^ tests to identify demographic and clinical factors significantly associated with mental health ED visits at 6-months post-discharge. Variables that were significantly associated with ED visits were then entered into a multivariable logistic regression model to predict ED utilization at the 6-months follow-up. Model fit was evaluated using the *χ*^2^ test, Cox and Snell *R*², and Nagelkerke *R*². Odds ratios (ORs) with 95% CIs were calculated to quantify the strength and direction of associations between predictor variables and ED visits. Statistical significance was set at *P*<.05. While ORs and *P* values provide estimates of association, the modest *R*² values signal that unmeasured variables contribute substantially to ED utilization risk, and findings should therefore be interpreted as indicative rather than determinative predictors.

## Results

### Univariate Analysis

Table 1 presents the findings from a univariate analysis exploring the relationship between ED visits at 6-months postdischarge and a range of demographic and clinical variables. Fourteen factors were assessed using *χ*^2^ tests (ie, study clusters, age, gender, ethnicity, education level, relationship status, employment status, housing status, primary mental health diagnosis, GAD-7, PHQ-9, WHO-5, BRS, prior 6-month ED visits), of which six demonstrated statistically significant associations. Participants younger than 25 years were more likely to report ED visits compared to older age groups. Individuals identifying as White people had higher rates of ED utilization relative to other ethnicities. Relationship status was also significant; single participants exhibited a greater likelihood of ED visits than those who were married, separated, divorced, or widowed. Employment status emerged as a notable factor, with unemployed individuals more frequently reporting ED use than those employed, retired, in school, or in other categories. Housing status also showed a significant association, with participants living with family or friends demonstrating the highest rates of ED visits at 6-months. Additionally, prior ED use within 6-months before discharge strongly predicted subsequent utilization, reinforcing the role of historical service use in forecasting future demand.

**Table 1.** Sociodemographic and clinical characteristics of participants at discharge from inpatient psychiatric units in Alberta, Canada.

Variables	Have had no ED^a^ visits at 6-months, n (%)	Have had ED visits at 6- months, n (%)	Total participants, n (%)	*χ* ^2^	*P* value
Age (years)				8.79	.01^b^
18‐25	253 (34.2)	142 (43.0)	395 (36.9)		
26‐40	257 (34.7)	108 (32.7)	365 (34.1)		
>40	230 (31.1)	80 (24.2)	310 (29.0)		
Ethnicity				9.85	.04^b^
White people	458 (61.9)	203 (61.5)	661 (61.8)		
Indigenous	60 (8.1)	38 (11.5)	98 (9.2)		
Black people	86 (11.6)	30 (9.1)	116 (10.8)		
Asian	77 (10.4)	44 (13.3)	121 (11.3)		
Mixed/Other	59 (8.0)	15 (4.5)	74 (6.9)		
Relationship status				10.0	.04^b^
Married/Partnered/Common-law	232(31.4)	79 (23.9)	311 (29.1)		
Single	420 (56.8)	213 (64.5)	633 (59.2)		
Separated or divorced	60 (8.1)	23 (7.0)	83 (7.8)		
Widowed	10 (1.4)	2 (0.6)	12 (1.0)		
Prefer not to say	18 (2.4)	13 (3.9)	31 (2.9)		
Employment status				19.90	<.001^b^
Employed	239 (32.3)	78 (23.6)	317 (29.6)		
Unemployed	362 (48.9)	206 (62.4)	568 (53.1)		
Student	64 (8.6)	23 (7.0)	87 (8.1)		
Retired	52 (7.0)	11 (3.3)	63 (5.9)		
Other	23 (3.1)	12 (3.6)	35 (3.3)		
Housing status				13.14	<.001^b^
Own home	162 (21.9)	49 (14.8)	211 (19.7)		
Rented accommodation	224 (30.3)	121 (36.7)	345 (32.3)		
Live with family or friends	315 (42.6)	132 (40.0)	447 (41.8)		
Couchsurfing/Shelter/Street/Other	38 (5.1)	28 (8.5)	66 (6.2)		
Prior 6-months ED visits				17.60	<.001^b^
No	345 (45.5)	110 (32.1)	455 (41.3)		
Yes	413 (54.5)	233 (67.9)	646 (58.7)		

a ED: Emergency Department.

b Statistically significant values.

### Logistic Regression Analysis Results

Six variables identified as statistically significant in the univariate analysis were included in a logistic regression model to examine predictors of ED visits 6-months post-discharge. This variable-selection strategy was chosen to maximize model parsimony and reduce the risk of overfitting, given the relatively modest sample size for regression analysis. To confirm robustness, an alternative model including all measured demographic and clinical variables was also tested. The pattern of results was consistent: age, employment, and prior ED use remained the strongest predictors, while nonsignificant predictors did not meaningfully improve model fit. We therefore report the more parsimonious model below. The model was statistically significant, *χ*²_31_=63.0, *P*<.001, indicating a good fit for distinguishing between individuals who did and did not access ED services after discharge. It accounted for 5.9% (Cox and Snell *R*²) to 8.3% (Nagelkerke *R*²) of the variance and correctly classified 68.1% (748/1098) participants. These values indicate that while the model improves classification compared to chance, it explains only a modest proportion of overall variance, underscoring the multifactorial and complex nature of ED utilization.

As shown in the model (Table 2), age was a significant predictor: individuals aged 26‐40 (OR 0.66, 95% CI 0.46‐0.95) and those over 40 (OR 0.58, 95% CI 0.37‐0.92) were significantly less likely to revisit the ED than those 25 and younger. The effect sizes suggest that being older reduced the odds of repeat ED use by approximately one-third to almost one-half, which is clinically meaningful given the high service burden of younger patients. Ethnicity also played a role, with participants identifying as “mixed or other” being less likely than White people to return to the ED (OR 0.52, 95% CI 0.28‐0.96). Although the overall effect was modest, this finding highlights possible protective factors among minority or mixed-ethnic groups that warrant further exploration. Employment status emerged as a strong predictor: unemployed individuals were significantly more likely to report ED visits than those employed (OR 1.66, 95% CI 1.18‐2.34). This effect size indicates that unemployment increased the likelihood of repeat ED visits by roughly two-thirds, a difference that is substantial in both clinical and policy contexts.

**Table 2.** Logistic regression model showing predictors of psychiatric emergency department visits within six months post-discharge among patients enrolled in TAU^a^, SMS, and SMS+PS^b^ groups.

Variables	B	SE	Wald statistic (*W*)	*df*	*P* value	Odds ratio (95% CI 95% for EXP(B)^c^)
Age (years)						
18-25	—^d^	—	6.77	2	.03^e^	—
26-40	−0.42	0.19	5.04	1	.03^e^	0.66 (0.46-0.95)
>40	−0.55	0.23	5.46	1	.02^e^	0.58 (0.37-0.92)
Ethnicity						
White people	—	—	10.292	4	.04^e^	—
Indigenous	0.11	0.24	2.89	1	.65	1.12 (0.70-1.78)
Black people	−0.41	0.24	3.07	1	.09	0.66 (0.41-1.07)
Asian	0.29	0.22	1.69	1	.19	1.33 (0.86-2.07)
Mixed/Other	−0.66	0.32	4,31	1	.04^e^	0.52 (0.28-0.96)
Employment status						
Employed			12.2	4	.02^e^	—
Unemployed	0.51	0.17	0.86	1	<.001^e^	1.66 (1.18-2.34)
Student	−0.1	0.3	0.11	1	.75	0.91 (0.50-1.64)
Retired	−0.07	0.39	0.03	1	.86	0.93 (0.43-2.02)
Other	0.09	0.47	0.03	1	.86	1.09 (0.43-2.74)
Housing status						
Own home			12,20	3	.01^e^	—
Rented accommodation	0.34	0.22	2.3	1	.13	1.41 (0.91-2.18)
Live with family or friends	−0.25	0.25	1	1	.32	0.78 (0.48-1.27)
Coach surfing/ Shelter/Street/Other	0.38	0.34	1.28	1	.26	1.46 (0.76-2.83)
Prior 6-month ED^f^ visits						
Had prior 6-month ED visits	0.89	0.44	4.12	1	.04^e^	2.45 (1.03-5.80)
Constant	–1.41	0.63	4.97	1	.03^e^	0.24^d^

a TAU: Treatment as usual

b PS: peer support.

c EXP(B): Exponentiated coefficient (odds ratio)

d Not applicable.

e Statistically significant *P* value.

f ED: Emergency department.

Housing status also contributed meaningfully to the model. Compared to individuals who owned their homes, those renting (OR 1.41, 95% CI 0.91‐2.18), living with family or friends (OR 0.78, 95% CI 0.48‐1.27), or in unstable housing conditions such as couch surfing or homelessness (OR 1.46, 95% CI 0.76‐2.83) showed varied but nonsignificant likelihoods of ED utilization. Although these estimates did not reach statistical significance, the directionality of effects is consistent with prior literature linking housing instability with increased ED reliance. Notably, prior ED visits in the 6-months before discharge significantly predicted future ED use (OR 2.45, 95% CI 1.03‐5.80), indicating that individuals with previous ED use were more than twice as likely to return post discharge. This strong association reinforces prior ED utilization as a key risk marker for repeat presentations and highlights its potential utility in risk stratification tools.

## Discussion

### Principal Findings

The present study evaluated factors associated with mental health emergency department visits among psychiatric patients within 6-months post discharge, emphasizing demographic and clinical predictors. The analysis revealed that age, ethnicity, employment status, housing status, and prior ED utilization significantly influenced ED revisits, offering critical insights for enhancing transitional care programs aimed at reducing recurrent ED visits among psychiatric patients.

Age was a significant predictor of ED use, with participants aged 26‐40 and those over 40 being significantly less likely to revisit the ED compared to those under 25 years. This finding is consistent with previous literature indicating that younger adults often face increased challenges following psychiatric discharge due to limited coping mechanisms, developmental transitions, and weaker social support systems [32,33]. Specific challenges include difficulties navigating health care systems independently, greater likelihood of unstable housing or employment, and higher prevalence of comorbid substance use, all of which may exacerbate reliance on emergency services. Younger adults may also be less engaged with primary care and community follow-up, increasing vulnerability to crisis presentations. From a service-planning perspective, these findings suggest that younger patients should be proactively flagged for additional follow-up, aligning with risk stratification models increasingly promoted in mental health policy.

Regarding ethnicity, the study found that participants identifying as mixed or other ethnic groups were significantly less likely to revisit the ED within 6-months post-discharge compared to White participants. This result contrasts with some prior studies that have reported higher service utilization among minority groups due to barriers in outpatient care and stigma [34,35]. However, it aligns with other literature suggesting that cultural resilience, extended family support, or reliance on community-based coping strategies may buffer the need for acute service use [36,37]. Cultural perceptions of mental illness, such as framing psychiatric distress through spiritual or community-based lenses, may also influence patterns of help-seeking. While these factors may lower ED utilization, they also risk masking unmet needs if formal care is underutilized. Understanding these protective mechanisms could inform the design of culturally tailored outreach and transitional supports.

Employment status significantly influenced ED utilization: unemployed individuals were 1.66 times more likely to return to the ED compared to those who were employed. This result aligns with previous findings suggesting that unemployment is associated with higher psychiatric symptom burden and greater use of acute care services, likely due to financial strain, reduced access to regular health care, and lack of routine [38,39]. These findings support the integration of vocational rehabilitation and employment-focused interventions into post-discharge plans to enhance stability and reduce emergency service dependence. Embedding employment support into transitional interventions such as SMS and peer support could increase both engagement and impact, particularly among high-risk unemployed groups.

Housing status contributed to the model, but there was no significant difference between any of the housing options and home ownership. Compared to individuals living with family or friends, participants who owned their homes exhibited a slightly lower likelihood of returning to the ED (OR 0.78; 95% CI 0.48‐1.27). In contrast, those living in rented accommodations (OR 1.41; 95% CI 0.91‐2.18) and those in unstable housing conditions, such as shelters, couch surfing, or homelessness (OR 1.46; 95% CI 0.76‐2.83), tended to show higher odds of ED visits within 6-months post-discharge. Although these differences did not reach statistical significance, the pattern suggests that housing stability may influence health care utilization. These findings align with existing literature indicating that insecure housing is associated with increased acute service use and mental health deterioration [20,21]. As such, future research should continue to investigate housing as a key social determinant of health and explore its potential role in discharge planning and ED prevention strategies. Housing should therefore be treated as a critical social determinant of psychiatric outcomes, and future intervention studies in Alberta should integrate housing supports alongside psychosocial care in discharge planning.

Perhaps most notably, prior mental health ED utilization strongly predicted future psychiatric ED revisits within 6-months of discharge. Participants with a history of ED attendance within the 6-months preceding their hospital discharge were significantly more likely to revisit the ED post-discharge compared to those without previous ED visits. This result aligns closely with existing literature emphasizing that previous emergency health care utilization is among the strongest indicators of subsequent ED reliance [40,41]. Frequent ED use often reflects unresolved or inadequately managed psychiatric and medical conditions, suggesting the presence of underlying chronic, complex health needs that persist despite hospitalization [6]. Addressing these unmet needs through coordinated care management and targeted intervention post-discharge is critical for reducing future emergency visits and improving overall mental health outcomes [6,35]. In practice, this supports the implementation of “high-utilizer” pathways that provide more intensive follow-up for patients with repeated ED use, a model already being piloted in some Canadian areas.

Although the logistic regression model was statistically significant and correctly classified 68.1% of cases, the amount of variance explained was modest (5.9% Cox and Snell *R*²; 8.3% Nagelkerke *R*²). This suggests that many important factors influencing ED use post-discharge were not captured in the model. Existing literature points to additional variables such as psychiatric diagnosis, substance use, medication adherence, co-occurring physical conditions, trauma history, and access to outpatient mental health services as critical influences on ED utilization [22,42,43]. Social determinants of health, including transportation barriers, social isolation, and stigma, may also play a significant role [44]. Rather than undermining the model, this limitation highlights the multifactorial nature of psychiatric crises and underscores the need for multi-level, biopsychosocial approaches to prediction.

Integrating across these findings, a picture emerges of intersecting social and clinical vulnerabilities: young age, unemployment, unstable housing, and prior ED use cluster together to identify a subgroup of patients at highest risk. These overlapping factors should not be addressed in isolation but through coordinated transitional care packages that combine psychosocial support, housing assistance, vocational services, and scalable interventions such as SMS and peer support.

The study findings have several clinical and policy implications. Discharge planning should incorporate risk stratification protocols that take into account factors such as age, housing status, and prior ED use. Tailored interventions, such as job placement assistance for unemployed individuals, supported housing programs for those experiencing instability, and enhanced follow-up for frequent ED users could help reduce avoidable ED visits. Integrating low-barrier, scalable solutions like supportive text messaging (SMS) and peer support services may also contribute to improved continuity of care. Prior studies have demonstrated the feasibility and acceptability of SMS-based interventions in postdischarge mental health care, offering a low-cost approach to maintaining patient contact, monitoring well-being, and providing timely support [45,46]. Peer support, while variably effective depending on delivery and patient engagement, remains a promising adjunct when implemented with appropriate oversight [45,46]. Future research should build on these findings by: (1) incorporating additional clinical and social predictors such as diagnosis severity, substance use, and medication adherence; (2) testing predictive models within culturally diverse subgroups to explore differential effects; and (3) evaluating how SMS and peer support interventions may moderate the risks identified in high-risk groups. Structured, mixed-methods designs may be especially useful in capturing patient perspectives on these mechanisms.

### Limitations

Several limitations must be acknowledged when interpreting the study findings. First, the modest variance explained by the logistic regression model (5.9% to 8.3%) suggests that additional unmeasured factors may contribute significantly to mental health ED revisits. These may include the severity of psychiatric conditions, comorbid medical disorders, medication adherence, or social determinants such as trauma history and stigma. In particular, clinical information such as symptom severity ratings, comorbid substance use, and adherence to prescribed treatments was not available in this dataset, which may have led to residual confounding and underestimation of the true strength of some predictors. Additionally, adherence and engagement with the SMS and peer support interventions were not tracked. As this was a secondary, intention-to-treat analysis, differences in exposure or participation (“dosage”) could not be assessed, which may have influenced post-discharge outcomes.

Second, our reliance on self-reported data introduces the potential for recall bias, which may affect the accuracy of participants’ responses, particularly regarding sociodemographic details and prior ED utilization. Although recall bias is difficult to quantify, prior studies suggest that retrospective reporting of service use may underestimate utilization, meaning that the observed associations with prior ED visits could in fact be conservative.

Third, although we captured a wide range of clinical and psychosocial variables, we did not assess whether participants had a regular general practitioner or how frequently they accessed primary care services. This is a critical limitation, as regular contact with a primary care provider has been shown to reduce unnecessary ED visits and support better outpatient continuity of care in psychiatric populations [47,48].

Fourth, the stepped-wedge design of the parent trial introduces potential clustering and time-related biases. Site-specific differences in resources, staffing, or policy changes occurring during the staggered rollout could have influenced both predictors and outcomes, limiting the ability to attribute findings solely to individual-level factors. While the design increased external validity by reflecting real-world conditions, it also constrained variable selection and confounder control.

Lastly, while our sample was drawn from multiple health care sites across Alberta, the generalizability of our findings may be limited to similar health care settings with comparable service delivery models and population demographics. Replication of this work in other provinces and internationally would help determine the transferability of identified predictors across health systems.

### Conclusion

This study underscores the complex and multifactorial nature of emergency department utilization following discharge from psychiatric inpatient care. Key predictors of post-discharge ED visits included younger age, White ethnicity, unemployment, housing instability, and prior ED use. These findings emphasize the importance of identifying high-risk individuals and implementing tailored, evidence-based interventions during the transition from hospital to community care. At the same time, the modest variance explained by the regression model indicates that these predictors capture only part of the risk profile, and results should be interpreted with caution rather than as definitive determinants of ED use.

Proactive strategies, such as risk stratification, targeted psychosocial support, and integration of digital tools, may reduce mental health ED recidivism, improve continuity of care, and alleviate pressure on emergency services. In particular, SMS and peer support represent low-cost, scalable approaches that could be embedded into discharge planning, especially for groups identified as higher risk. While our study was not designed to evaluate intervention effects directly, situating predictors within this transitional care context strengthens the clinical and policy relevance of the findings.

It is important to emphasize that the primary aim of this secondary analysis was to identify predictors of psychiatric ED visits, not to assess intervention efficacy. Findings therefore apply to patients randomized to TAU, SMS, or SMS+ PS, and should not be generalized to all psychiatric patients presenting to EDs. As such, the results are best viewed as hypothesis-generating and useful for refining risk stratification and tailoring transitional interventions in Alberta’s mental health system.

Given the limited predictive power of the current model and the omission of several important variables, future research should expand predictor sets to include illness severity, comorbidities, treatment adherence, and outpatient engagement. Cross-provincial and international replication will also be essential to establish generalizability beyond the Alberta health system.

## Abbreviations

BRS: Brief Resilience Scale
ED: emergency department
GAD-7: Generalized Anxiety Disorder 7-item scale
ICC: intraclass correlation
ITT: intention-to-treat
OR: odds ratio
PHQ-9: Patient Health Questionnaire-9
PS: peer support
TAU: treatment as usual
WHO-5: World Health Organization-Five Well-Being Index
